# A Case of Malignant Pheochromocytoma Detected during Fertility Treatment

**DOI:** 10.1155/2014/646743

**Published:** 2014-02-05

**Authors:** Kazuhisa Hagiwara, Itsuto Hamano, Ayumu Kusaka, Hiromi Murasawa, Noriko Tokui, Kengo Imanishi, Akiko Okamoto, Hayato Yamamoto, Atsushi Imai, Shingo Hatakeyama, Takahiro Yoneyama, Yasuhiro Hashimoto, Takuya Koie, Chikara Ohyama

**Affiliations:** Department of Urology, Hirosaki University Graduate School of Medicine, Hirosaki, Aomori 036-8562, Japan

## Abstract

We report a case of malignant pheochromocytoma in a 35-year-old Japanese woman during fertility treatment, successfully treated with surgical excision. The patient recovered without any postoperative problems, and plasma catecholamine levels normalized. At present, 18 months after the operation, there are no signs of relapse.

## 1. Introduction

Although the incidence of malignant tumors during fertility treatment with ovulation-inducing drugs has been described in several reports, many authors state that in the short-term, ovulation-induction treatment may not be a risk factor for ovarian cancer. Herein we describe a case of malignant pheochromocytoma detected during fertility treatment.

## 2. Case Report

We evaluated a 35-year-old woman with no medical or family history of endocrine disease. She underwent surgical removal of the benign breast tumor at the age of 22 and underwent a surgical procedure for a uterine myoma at the age of 32. She started visiting the Department of Obstetrics and Gynecology at our hospital for fertility treatment in 2009 (31 years of age). In January 2012, a tumor of 10 cm diameter was identified on the inferior side of the right hepatic lobe via abdominal ultrasound screening. Malignant pheochromocytoma was diagnosed using imaging (CT, MRI) and endocrine testing, and consequently the patient was referred to our department for a surgical procedure. The subjective symptoms were as follows: no headache, no pallor of the face, no palpitations, and a sudden rise in temperature. There was no hypertension. The patients' characteristics recorded at the time of hospital admission were as follows: height 149.8 cm, weight 57.6 kg, BMI 25.7, blood pressure 110/65, heart rate 75 bpm, and body temperature 36.4°C. The abdominal tumor was not palpable. Biochemical examination of blood showed fractionated plasma catecholamine levels as follows: adrenaline <5 ng/mL (0–100), noradrenaline 809 ng/mL (100–450), and dopamine 8 ng/mL (0–20); thus, only the noradrenaline level was elevated. Fractionated urinary catecholamines were as follows: adrenaline 10 *μ*g/day (1.5–4.3), noradrenaline 568 *μ*g/day (3.4–26.9), and dopamine 685 *μ*g/day (365–961); only the noradrenaline level was elevated.

**Figure 1:**
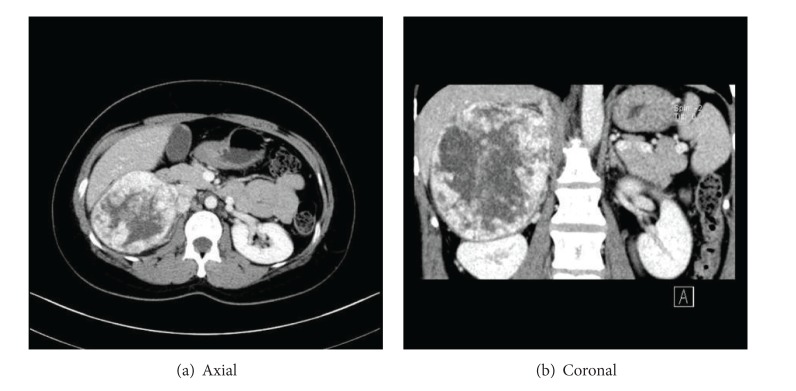
CT scan of the abdomen showing a mass in the right adrenal gland.

Diagnostic imaging by means of abdominal CT revealed a tumor 11 × 10 cm in size on the inferior side of the liver, which displaced the right kidney (Figure 1). Swelling was also observed in the lymph nodes around the inferior vena cava. MRI revealed an adrenal tumor exhibiting low signal intensity on T1-weighted imaging and high signal intensity on T2-weighted imaging. At the same time, MIBG (metaiodobenzylguanidine) accumulation in the right adrenal tumor was revealed in the scintigraphy images.

**Figure 2:**
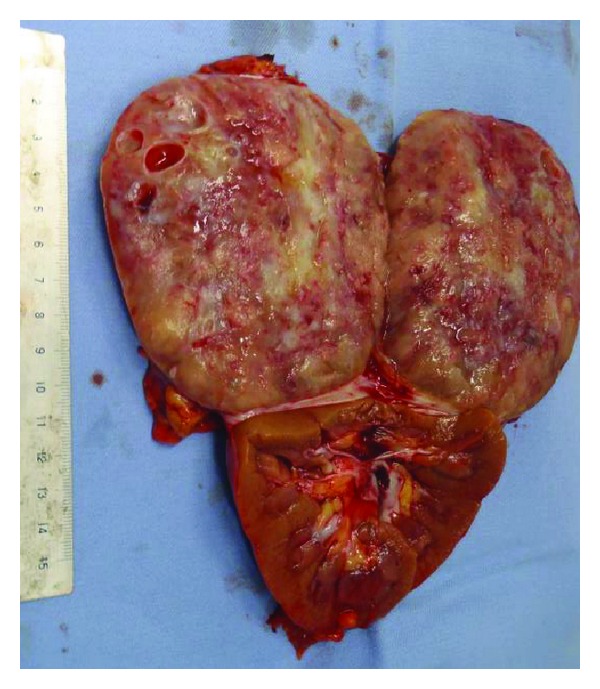
Macroscopic finding of surgical specimen.

**Figure 3:**
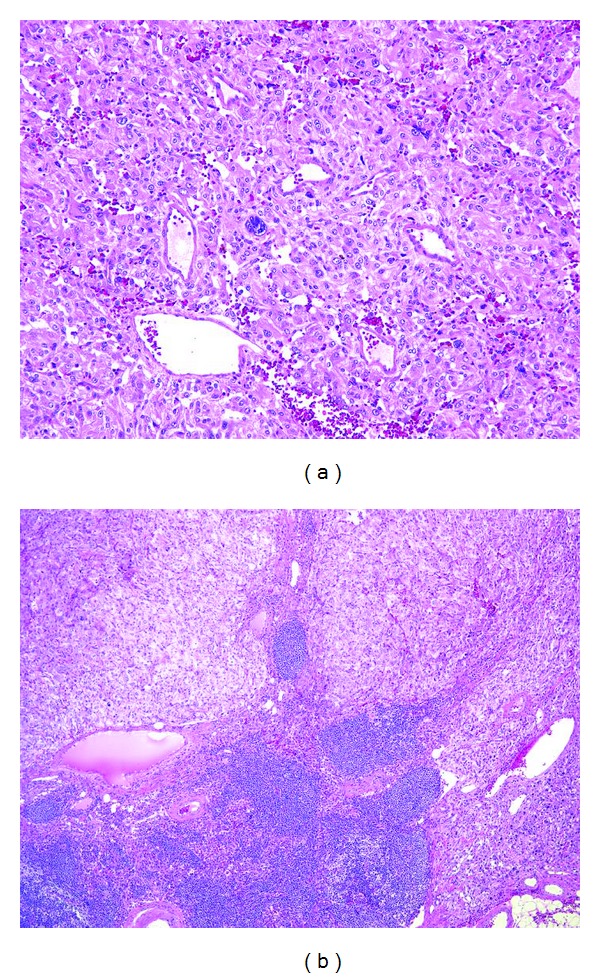
(a) Microscopically, polygonal basophilic atypical cell proliferated in rich vascular network. There were mitotic cells and multinuclear cells (HE, original magnification ×100). (b) Tumor cell involved lymph node (HE, original magnification ×40).

Right pheochromocytoma was diagnosed, and the patient underwent surgery in May 2012. Because CT led to the suspicion of adhesion to surrounding tissue, laparotomy was conducted. A skin incision was made from the xiphoid process to the right hypochondriac region. The tumor had fused to the right hepatic lobe, inferior vena cava, and the right kidney. Because adhesion to the right kidney was strong, a combined right nephrectomy was performed. The swollen lymph nodes around the inferior vena cava were also surgically removed (total duration of the surgical procedure: 5 h and 4 min; blood loss: 6,090 mL). The resected specimen weighed 600 g (Figure 2). Pathological examination revealed a large number of nuclear fissions in the tumor cells and lymph node metastases, which led to the diagnosis of malignant pheochromocytoma with a lymph node metastasis [1] (Figure 3). The patient recovered without any postoperative problems, and plasma catecholamine levels normalized. Examination by imaging revealed no residual tumor, and an adjuvant therapy was not administered because the patient had plans to conceive. The patient is continuing fertility treatment. The patient is currently adhering to strict follow-up as an outpatient; at present, 18 months after the procedure, there are no signs of relapse.

## 3. Discussion

Pheochromocytoma is associated with the following syndromes: type 2 multiple endocrine neoplasia (MEN2), Von Hippel-Lindau Disease (VHL), and neurofibromatosis type 1 (NF1) [2]. Although 17% of these tumors are malignant, pathologically there are no molecular or histologic markers, and therefore when metastatic lesions are discovered postoperatively or when unresectable lesions are found, many patients receive a diagnosis of malignant pheochromocytoma or malignant paraganglioma [3]. In the present case, there is no relevant family history that could cause pheochromocytoma, and therefore it can be considered sporadic.

Although it has been debated whether ovulation-inducing drugs increase the incidence of ovarian, uterine, and breast cancers, a firm conclusion remains unclear. Many reports indicate that the incidences of ovarian, uterine, and breast cancers are not increasing [4–7], but it has also been reported that, in actual clinical practice, ovarian cancer rapidly progresses when detected during fertility treatment with ovulation-inducing drugs [8]. We were unable to find any other articles where malignant pheochromocytoma was detected during fertility treatment, and so it is unclear whether or not the ovulation-inducing drugs caused the malignant pheochromocytoma. The possible causal link seems to warrant some basic research.
